# ASSOCIATION BETWEEN AFFECTIVE TEMPERAMENT AND MORBID OBESITY IN BARIATRIC SURGERY CANDIDATES: A CASE-CONTROL STUDY

**DOI:** 10.1590/0102-67202025000015e1884

**Published:** 2025-05-23

**Authors:** Alexandre Karam Joaquim MOUSFI, Sivan MAUER, Paulo Afonso Nunes NASSIF, Marcos Fabiano SIGWALT, Ronaldo Mafia CUENCA, Orlando Jorge Martins TORRES

**Affiliations:** 1 Hospital Universitário Evangélico Mackenzie - Curitiba (PR), Brazil;; 2 Faculdade Evangélica Mackenzie do Paraná - Curitiba (PR), Brazil;; 3 Universidade de Brasília, Hospital de Brasília, Surgical Clinic Center - Brasília (DF), Brazil;; 4 Universidade Federal do Maranhão, Department of Gastrointestinal Surgery - São Luis, (MA), Brazil.

**Keywords:** Temperament, Mood Disorders, Obesity, Morbid, Bariatric Surgery, Temperamento, Transtornos do Humor, Obesidade Mórbida, Cirurgia Bariátrica

## Abstract

**BACKGROUND:**

Affective temperaments are part of the spectrum of mood disorders and comprise the concepts of hyperthymia, dysthymia and cyclothymia. Numerous studies have demonstrated a strong relationship between obesity and mood disorders.

**AIMS:**

The objective of the present study was to evaluate the frequency of affective temperaments in morbidly obese individuals and controls and to establish a possible association between affective temperaments and morbid obesity.

**METHODS:**

The study evaluated 106 cases (morbidly obese) and one hundred controls (non-obese). To assess affective temperaments, the Temperament Evaluation in Memphis Pisa and San Diego - Rio de Janeiro TEMPS-Rio de Janeiro scale was applied. Depressive symptoms were assessed using the Hamilton Depression Rating Scale, anxiety symptoms using the Hamilton Anxiety Rating Scale and manic symptoms using the Young Mania Rating Scale. For univariate and multivariate analysis, logistic regression models were adjusted.

**RESULTS:**

The presence of at least one affective temperament was 74.5% in the morbidly obese group and 63% in the non-obese group. When comparing the two groups, the statistical analysis of the age subgroup of individuals aged 50 years or over showed an odds ratio of 2.56 (1.07-6.09) for hyperthymic temperament.

**CONCLUSIONS:**

In the age group of 50 years or more, cases of morbid obesity are significantly more likely (2.56 times) to occur in individuals with a hyperthymic temperament. Among the three types of affective temperaments evaluated, only hyperthymia could be a risk factor for morbid obesity.

## RESUMO

**RACIONAL:**

Os temperamentos afetivos fazem parte do espectro dos transtornos do humor e compreendem os conceitos de hipertimia, distimia e ciclotimia. Vários estudos demonstraram uma forte relação entre obesidade e transtornos do humor.

**OBJETIVOS:**

O objetivo do presente estudo foi avaliar a frequência de temperamentos afetivos em indivíduos com obesidade mórbida e controles e estabelecer uma possível associação entre temperamentos afetivos e obesidade mórbida.

**MÉTODOS:**

O estudo avaliou 106 casos (obesos mórbidos) e 100 controles (não obesos). Para avaliar os temperamentos afetivos foi aplicada a escala TEMPS-Rio de Janeiro. Os sintomas depressivos foram avaliados pela Escala de Avaliação de Depressão de Hamilton, os sintomas de ansiedade pela Escala de Avaliação de Ansiedade de Hamilton e os sintomas maníacos pela Escala de Avaliação de Mania de Young. Para análise univariada e multivariada foram ajustados modelos de Regressão Logística.

**RESULTADOS:**

A presença de pelo menos um temperamento afetivo foi de 74,5% no grupo de obesos mórbidos e de 63% no grupo de não obesos. Na comparação entre os dois grupos, a análise estatística do subgrupo etário dos indivíduos com 50 anos ou mais mostrou uma razão de chances de 2,56 (1,07-6,09) para o temperamento hipertímico.

**CONCLUSÕES:**

Na faixa etária de 50 anos ou mais, os casos de obesidade mórbida são significativamente mais prováveis (2,56 vezes) de ocorrer em indivíduos com temperamento hipertímico. Entre os três tipos de temperamentos afetivos avaliados, apenas a hipertimia poderia ser fator de risco para obesidade mórbida.

**Central Message**

Obesity is a complex and multifactorial disease that negatively interferes with the body’s physiological functions and increases the risk of conditions such as diabetes, cardiovascular diseases, premature death, some types of cancer, musculoskeletal and psychiatric diseases. Numerous studies have demonstrated a strong relationship between obesity and psychiatric illnesses, especially mood disorders.

**Perspectives**

It is recommended that all morbidly obese patients in the preoperative period be properly evaluated for psychiatric conditions, with the aim of previously identifying conditions that may interfere with the outcome of the procedure. Adequate diagnosis and treatment of affective temperament, especially hyperthymic temperament, can be extremely important in the preoperative evaluation of patients who are candidates for bariatric surgery, since the symptoms presented can directly interfere with the medium and long-term results of the surgical procedure. Cases of morbid obesity have a significantly greater chance (2.56 times) of occurring in individuals with a hyperthymic temperament, compared to individuals who do not have this temperament, in the age group of 50 years or more. Among the three types of affective temperaments evaluated, only hyperthymia could be a risk factor for morbid obesity.

## INTRODUCTION

The concept of temperament was originally described in ancient Greece in the Hippocratic period, explaining pathological emotions based on the theory of the four humors^3^. In the 19^th^ century, Emil Kraepelin described four fundamental states -depressive, manic, cyclothymic and irritable -, which he considered subclinical forms and possible precursors of affective disorders, beginning in adolescence^23^ ^,^ ^54^. However, Ernst Kretschmer was the one who best systematized the concept of temperament, describing hyperthymia for the first time^22^ ^,^ ^51^. Affective temperaments are currently understood as part of the spectrum of mood disorders, based on family, genetic and clinical studies^19^ ^,^ ^30^. Temperaments have a genetic and biological origin, as well as a high heritability^19^. Temperaments can also be seen as precursors, risk factors or even mediators of different clinical manifestations of mental illnesses^17^. The three main types of affective temperaments are hyperthymia, dysthymia and cyclothymia. Hyperthymia presents as a mild and chronic manic state. Such individuals have a higher level of energy, less need for sleep, are very sociable, have an increased sexual drive and are more prone to the abuse of psychoactive substances^1^ ^,^ ^2^. Dysthymia consists of mild and persistent depressive symptoms. The individual has a low energy level, has a greater need for sleep, reduced sexual drive and a greater inclination to have worries related to personal failures^3^ ^,^ ^29^. Cyclothymia is a constant alternation between manic and depressive symptoms, lasting a day or a few days. These people present characteristics such as affective lability, anger and irritability, in addition to attentional difficulties^3^.

Obesity is a complex and multifactorial disease that negatively interferes with the body’s physiological functions and increases the risk of conditions such as diabetes, cardiovascular diseases, premature death, some types of cancer, musculoskeletal and psychiatric diseases^6^ ^,^ ^12^ ^,^ ^33^. The prevalence of overweight and obesity has doubled since the 1980s, now reaching almost a third of the world’s population, making it an important public health problem^12^ ^,^ ^27^. The prevalence of overweight is lower in young adult women and higher in women over 45 years of age^12^. The prevalence of obesity is higher in women, in all age groups, and increases with age^12^. Bariatric surgery is the most effective long-term treatment for morbid obesity refractory to medical treatment, especially with comorbid diabetes^9^.

Numerous studies have demonstrated a strong relationship between obesity and psychiatric illnesses, especially mood disorders^11^ ^,^ ^20^ ^,^ ^24^ ^,^ ^38^ ^,^ ^41^ ^,^ ^42^. This relationship appears to have a bidirectional nature^6^. The most common psychiatric conditions in overweight and obese patients are mood disorders, binge eating disorder and anxiety disorders^7^ ^,^ ^14^ ^,^ ^34^ ^,^ ^45^ ^-^ ^47^.

The objective of the present study was to evaluate the frequency of the three main types of affective temperaments (dysthymia, cyclothymia and hyperthymia) in morbidly obese patients and in controls without a diagnosis of obesity and to establish a possible association between affective temperaments and morbid obesity in candidate patients to bariatric surgery.

## METHODS

### Design and subjects

A cross-sectional case-control study comparing patients with morbid obesity, and non-obese controls. The control group consisted of a hundred patients without a diagnosis of obesity, followed in outpatient services of endocrinology (25 individuals), rheumatology (42 individuals) and ophthalmology (33 individuals) at Mackenzie Evangelical University Hospital, located in Curitiba, state of Paraná, Brazil. The case group comprised 106 patients diagnosed with morbid obesity, candidates for surgical treatment, followed at the Bariatric and Metabolic Surgery outpatient clinic of the same hospital. The evaluation took place from September 2022 to May 2023.

*Inclusion criteria*

Controls:

1. Body mass index (BMI) less than 30 kg/m²;
2. Over 18 years of age;
3. Collaborative with the application of tests;
4. Signed the informed consent.

Cases:

1. BMI greater than or equal to 40 kg/m² or BMI greater than or equal to 35 kg/m² associated with comorbidities (diabetes mellitus, arterial hypertension, dyslipidemia, coronary disease, osteoarthritis, sleep apnea, etc.);
2. In pre-operative monitoring for bariatric surgery;
3. Over 18 years of age;
4. Collaborative with the application of tests;
5. Signed the informed consent.

*Exclusion criteria*

For both groups, the following exclusion criteria were used:

1. Refusal to collaborate with the proposed tests;
2. Functional or sensory impairment that prevented the assessment;
3. Being pregnant;
4. Having previously undergone bariatric surgery.

This study followed the standards established by the National Council of Health and was approved by the ethics committee of Mackenzie Evangelical College of Paraná (number 5.804.715).

### Rating scales

A questionnaire was initially applied with personal data, sociodemographic questions and information on previous medical, psychiatric and family history.

Affective temperaments were assessed with the Brazilian version of the Temperament Scale of Memphis, Pisa, Paris and San Diego Autoquestionnaire (TEMPS-A) self-report questionnaire^54^. The Brazilian version has six subscales, i.e. a dysthymic, cyclothymic, hyperthymic, irritable, worrying and anxious temperament, measured by 45 items. Each item represents a statement which the patient has to rate (true or false) according to whether it applies to him or her. Only the first three subscales were used in the present study. Each of these subscales consists of eight items; the result of each subscale multiplied by 12.5 yields a total score that ranged between 0 and 100. A temperament was considered present when at least 75% of one sub-scale was confirmed^51^. Although patients can score positive for more than one type, overlap is generally minimal when using the 75% cut-off.

Depressive symptoms were assessed using the validated Portuguese version of the Hamilton Depression Rating Scale (HAM-D)^25^. Anxiety symptoms, using the validated Portuguese version of the Hamilton Anxiety Rating Scale (HAM-A)^26^. Manic symptoms, using the Portuguese version of the Young Mania Rating Scale (YMRS)^50^.

### Statistical analysis

The data were analyzed using the Stata/SE v.14.1 computer program (StataCorp LP, USA). The results of quantitative variables were described by mean and standard deviation. Categorical variables were described by absolute frequency and percentage. For the univariate and multivariate analysis of factors associated with obesity, logistic regression models were adjusted, and the Wald test was used to assess the significance of the variables. The estimated measure of association was the odds ratio with 95% confidence intervals. The normality condition of quantitative variables was assessed using the Kolmogorov-Smirnov test.

Statistical analysis included the description of the sample according to age, education, weight, height, BMI, gender, marital status, and ethnicity. Current and previous psychiatric characteristics, family history and clinical history were also evaluated.

The sample was stratified into two groups: case group (with a diagnosis of morbid obesity) and control group (without a diagnosis of obesity). In addition, age stratification was carried out into less than 50 years of age and 50 years or more.

## RESULTS

### Study sample

The analysis was carried out based on data from 206 patients, 106 of whom were morbidly obese (case group) and one hundred were non-obese (control group). Table 1 shows the sociodemographic characteristics of the studied population, separated by groups.

**Table 1:** Sociodemographic characteristics.

Characteristic/Classification	Sample					
	Total (n=206)		Control (n=100)		Case (n=106)	
Age (years) (mean±SD)	206	48.1±13.8	100	53.6±14.3	106	42.9±11.0
Age (years) (%)						
18-49	109	52.9	36	36	73	68.9
50-84	97	47.1	64	64	33	31.1
Education level (years) (mean±SD)	206	9.9±3.7	100	9.7±4.0	106	10.1±3.3
Weight (kg) (mean±SD)	206	94.9±30.4	100	69.6±11	106	118.7±22.5
Height (m) (mean±SD)	206	1.63±0.09	100	1.64±0.1	106	1.63±0.09
BMI (kg/m^2^) (mean±SD)	206	35.6±10.7	100	26.0±2.9	106	44.7±6.6
Gender (%)						
Female	152	73.8	69	69.0	83	78.3
Male	54	26.2	31	31.0	23	21.7
Marital status (%)						
Married	104	50.5	49	49.0	55	51.9
Single	42	20.4	21	21.0	21	19.8
Stable union	23	11.2	6	6.0	17	16.0
Separated/Divorced	21	10.2	15	15.0	6	5.7
Widower	16	7.8	9	9.0%	7	6.6
Ethnicity (%)						
White	145	70.4	75	75.0	70	66.0
Brown	38	18.4	18	18.0	20	18.9
African American	23	11.2	7	7.0	16	15.1

SD: standard deviation; BMI: body mass index ; kg: kilogram.

Some psychiatric diagnosis was reported by 33.5% of individuals evaluated in the total sample. Mood disorders (depression and bipolar disorder) were the most prevalent, present in 19%. Next, we had anxiety, with 13.6%. In the group of patients with morbid obesity, 33% reported having some psychiatric diagnosis. The percentage reporting a diagnosis of mood disorders was 22.6% and anxiety disorders, 10.4%.

Around 20% of the individuals evaluated reported undergoing some current psychiatric drug treatment and 13.1%, psychotherapy. The case group presented a greater number of individuals in psychiatric treatment (23.6 x 18.0%) and psychotherapy (17.9 x 8.0%) compared to the control group. Previous suicide attempts were more frequent in the control group (10%) than in the case group (5.7%).

A family history of some psychiatric illness was reported by 56.8% of patients in general, with mood disorders (depression and bipolar disorder) being the most prevalent (41.2%). Alcohol abuse and dependence appear in 19.9% and anxiety disorders in 19.4% of the general sample. A family history of suicide was present in 5.3% of the total number of individuals.

A history of clinical (non-psychiatric) illnesses were present in 79.1% of the general sample. In the group of patients with morbid obesity, 79.2% of individuals had some clinical comorbidity. The most prevalent diseases were hypertension (45.2%), diabetes mellitus (31.3%), dyslipidemia (22.6%) and thyroidopathy (21.7%).

### Rating scales

The presence of at least one affective temperament (dysthymic, cyclothymic or hyperthymic) was 74.5% in the morbidly obese group and 63% in the non-obese group, with some individuals meeting criteria for more than one temperament.

Table 2 shows the results found with the application of the diagnostic scales.

**Table 2:** Rating scales.

Scale/Classification	Group			
	Control (n=100) (%)		Case (n=106) (%)	
TEMPS-A - Dysthymia				
Negative (<75%)	93	93.0	96	90.6
Positive (=75%)	7	7.0	10	9.4
TEMPS-A - Cyclothymia				
Negative (<75%)	79	79.0	83	78.3
Positive (=75%)	21	21.0	23	21.7
TEMPS-A - Hyperthymia				
Negative (<75%)	52	52.0	41	38.7
Positive (=75%)	48	48.0	65	61.3
Hamilton Depression Rating Scale (HAM-D)				
Normal	54	54.0	60	56.6
Mild depression	39	39.0	41	38.7
Moderate depression	5	5.0	4	3.8
Severe depression	2	2.0	1	0.9
Hamilton Anxiety Rating Scale (HAM-A)				
Mild anxiety	89	89.0	98	92.5
Moderate anxiety	7	7.0	4	3.8
Severe anxiety	4	4.0	4	3.8
Young Mania Rating Scale				
No mania	100	100	106	100

TEMPS-A: Temperament Scale of Memphis, Pisa, Paris and San Diego Autoquestionnaire.

### Association between affective temperament and morbid obesity

When comparing the two groups through univariate analysis, an odds ratio of 1.38 was found in relation to dysthymic temperament, with a 95% confidence interval of 0.51-3.79. Assessing the cyclothymic temperament, an odds ratio of 1.04 (0.54-2.03) was found. In the analysis of hyperthymic temperament, the odds ratio was 1.72 (0.99-2.99), suggesting that morbidly obese individuals are 72% more likely to have hyperthymia compared to non-obese individuals.

The sample was stratified into two age groups (under 50 years old and 50 years old or over). Table 3 shows the data found in this evaluation.

**Table 3:** Association between affective temperaments and morbid obesity in individuals under 50 years of age (Univariate Logistic Regression Model and Wald test, p<0.05).

Variable/Classification	Group				p*	OR (IC95%)
	Control		Case			
	n	%	n	%		
Dysthymia						
Negative (<75%)	34	94.4	66	90.4		
Positive (=75%)	2	5.6	7	9.6	0.762	1.03 (0.85-1.25)
Cyclothymia						
Negative (<75%)	26	72.2	53	72.6		
Positive (=75%)	10	27.8	20	27.4	0.937	0.99 (0.86-1.15)
Hyperthymia						
Negative (<75%)	14	38.9	29	39.7		
Positive (=75%)	22	61.1	44	60.3	0.933	0.97 (0.43-2.19)

*OR: odds ratio; IC: information coefficient.

The statistical analysis of the age group of individuals aged 50 years or over, presented in Table 4, showed an odds ratio of 2.56 (1.07-6.09) for hyperthymic temperament.

**Table 4:** Association between affective temperaments and morbid obesity in individuals aged 50 years and over (Univariate Logistic Regression Model and Wald test, p<0.05).

Variable/Classification	Group				p*	OR (IC95%)
	Control		Case			
	n	%	n	%		
Dysthymia						
Negative (<75%)	59	92.2	30	90.9		
Positive (=75%)	5	7.8	3	9.1	0.268	0.89 (0.72-1.1)
Cyclothymia						
Negative (<75%)	53	82.8	30	90.9		
Positive (=75%)	11	17.2	3	9.1	0.685	0.97 (0.81-1.2)
Hyperthymia						
Negative (<75%)	38	59.4	12	36.4		
Positive (=75%)	26	40.6	21	63.6	0.034	2.56 (1.07-6.1)

*OR: odds ratio; IC: information coefficient.

For multivariate statistical analysis, the covariates that showed the greatest percentage difference between the two groups were used: age, gender, marital status, ethnicity, and diagnosis of anxiety disorder.

It was investigated whether, for the age group of 50 years or more, hyperthymia is a factor independently associated with obesity, considering the factors gender, ethnicity, marital status and diagnosis of anxiety disorder. To this end, initially, a univariate model was adjusted for each of these variables. Next, a multivariate model for obesity was adjusted, including hyperthymia as an explanatory variable and, as covariates, the variables that presented p<0.10 in the univariate analysis (gender and marital status). The results of the multivariate analysis indicate that, for those aged=50 years, regardless of gender and marital status, having hyperthymia tends to be associated with obesity (p=0.082).

## DISCUSSION

Our results indicate that individuals with morbid obesity have a high rate of affective temperaments (dysthymia, cyclothymia or hyperthymia), especially hyperthymia. The 74.5% prevalence of affective temperaments in the morbidly obese group was higher than the control group and much higher than that found in the general population, with rates varying from 12.9 to 20%^48^ ^,^ ^49^. This finding is in line with a single previous study using a similar methodology that found a prevalence of affective temperaments of almost 65% in morbidly obese individuals and of 37% in the control group, a statistically significant difference^5^.

Among the types of temperaments evaluated, the most prevalent among morbidly obese people was the hyperthymic temperament, present in 61.3% of patients. A previous study indicated a higher prevalence of cyclothymic temperament in this population, in relation to dysthymic and hyperthymic temperaments^5^. Compared to the control group, we found a difference (61.3% x 48%) that was not statistically significant. There was also no significant difference in the comparison of dysthymia and cyclothymia between the case and control groups.

Statistical analysis indicated a significant association between hyperthymic temperament and morbid obesity in the age subgroup of individuals aged 50 years and over. This association indicates that hyperthymia can be understood as a risk factor for morbid obesity in this age group, increasing the risk by 2.56 times. The literature points to this association only with the cyclothymic temperament^5^. Understanding that hyperthymia and cyclothymia are part of the bipolar spectrum, our finding is comparable with the results of a previous study that indicated a prevalence of 89% of bipolar spectrum diagnoses in morbidly obese patients seeking surgical treatment^4^ ^,^ ^18^.

The main interpretation of this result would be that temperaments with manic symptoms, hyperthymia and cyclothymia present clinical characteristics such as impulsivity, anxiety and addictive behaviors, manifesting themselves in a chronic course and favoring dysfunctional eating behaviors^2^ ^,^ ^3^ ^,^ ^11^ ^,^ ^18^ ^,^ ^31^, could contribute to the onset of morbid obesity in the long term.

One hypothesis to explain the association between hyperthymic temperament and morbid obesity only in the group of individuals aged 50 or over would be that morbid obesity may take a few decades to establish itself. Therefore, in younger individuals, such an association would not be found. The literature shows that the prevalence of obesity increases with age^12^.

Another plausible hypothesis, taking into account the higher percentage of women in the morbidly obese group (78.3%), would be the contribution of menopause to weight gain, increasing the risk of patients over 50 years of age developing morbid obesity^39^. Studies indicate that the prevalence of obesity is higher in women^12^. It is also known that a higher proportion of women in the morbidly obese population seeking surgical treatment, found in our study, is in line with data from the literature^4^.

The estimation at the time of assessment of depressive conditions using the HAM-D scale and of anxious conditions using the HAM-A scale did not indicate a significant difference in the comparison between the case group and the control group. This finding is contrary to that presented in the literature, which suggests higher rates of depressive and anxiety disorders in morbidly obese people, in cross-sectional assessments, compared to the general population^16^. The assessment of mania symptoms using the YMRS was negative in all subjects who participated in this study.

In the interviews, when we asked about the presence of any psychiatric diagnosis throughout life, we found a percentage of 33% in the group of morbidly obese people. Most studies that evaluated the lifetime prevalence of psychiatric illnesses in morbidly obese candidates for bariatric surgery indicated high rates in relation to the general population. Rates ranged from 36.8 to 72.6% in analyses carried out in the USA^28^ ^,^ ^36^ ^,^ ^43^, Europe^31^ ^,^ ^37^ and Brazil^16^. The prevalence of psychiatric illnesses in the general population ranges from 4.3 to 29.6%^15^. A possible bias in the percentage found in the present study could be the underdiagnosis of psychiatric illnesses in Brazil, often associated with patients’ difficulty in accessing specialized psychiatric services.

The most prevalent conditions among morbidly obese people were mood disorders (major depression and bipolar disorder), present in 22.6%. In second place we find anxiety disorders, at 10.4%. This higher prevalence of mood conditions coincides with the literature^11^ ^,^ ^16^.

Distinguishing between major depression and bipolar disorder in a retrospective analysis is relatively difficult, due to the difficulty of accessing previous hypomanic episodes. Therefore, many patients with bipolar II disorder or subsyndromal bipolar disorder are mistakenly diagnosed with major depression^8^. Previous studies suggest an association between episodes of major depression in obese patients and the subsequent diagnosis of bipolar disorder or bipolar spectrum disorders^13^ ^,^ ^40^.

Several studies show a frequent association between mood disorders and compulsive eating behaviors (binge eating disorder or bulimia nervosa), with higher rates of mood disorders in obese people with compulsive eating compared to those without such eating behavior^31^ ^,^ ^33^ ^,^ ^47^ ^,^ ^52^ ^,^ ^53^.

Knowing that many symptoms of hyperthymic temperament are associated with dysfunctional eating behaviors that can increase the risk of morbid obesity, early diagnosis of an affective temperament and its appropriate treatment could reduce the risk of developing morbid obesity in the long term^32^. Prospective studies are needed to evaluate this hypothesis.

Evidence suggests that preoperative psychiatric conditions are associated with worse postoperative outcomes, such as insufficient weight loss, surgical complications, and psychosocial problems^24^ ^,^ ^32^ ^,^ ^44^. Around 20 to 30% of patients undergoing bariatric surgery experience insufficient weight loss or weight regain in the first few years after surgery^44^. Therefore, in individuals with morbid obesity and seeking surgical treatment, the identification and appropriate approach to hyperthymic temperament preoperatively could reduce the chance of complications and unsatisfactory postoperative results. Controlled prospective studies are needed to confirm this hypothesis.

Several risk factors have been associated with weight regain after bariatric surgery, such as binge eating, emotional eating, loss of control and disinhibition when eating, and anxiety^10^ ^,^ ^21^. As these factors may be consequences of symptoms of hyperthymic temperament, the appropriate approach could minimize the risk of weight regain. Studies evaluating this hypothesis are needed to confirm it.

It is recommended that all morbidly obese patients in the preoperative period be properly evaluated for psychiatric conditions^35^, with the aim of previously identifying conditions that may interfere with the outcome of the procedure.

Adequate diagnosis and treatment of affective temperament, especially hyperthymic temperament, can be extremely important in the preoperative evaluation of patients who are candidates for bariatric surgery, since the symptoms presented can directly interfere with the medium and long-term results of surgical procedure^24^.

The present study has some limitations that must be considered. One of them would be the small sample size of obese individuals, which reduces the chance of finding significance in the statistical analysis carried out. The statistical evaluation of the multivariate analysis in the age group of 50 years and over was possibly compromised due to the sample size.

The generalization of the sample is compromised considering that the rate of women evaluated was much higher than that of men and the percentage of white individuals was much higher than that of brown or African Americans. Another point is that the sample evaluated was convenient within outpatient services in a single university hospital, preventing generalization to other cities or regions of Brazil.

The assessment of previous psychiatric diagnoses and family psychiatric history was based on the patient’s report, raising possible questions about the validity of the information reported.

In the study, we did not assess the impact of possible side effects of medications in use on increased appetite, possibly contributing to the manifestation of obesity.

No detailed assessment was carried out on the use of stimulant psychoactive substances, which could mimic symptoms present in the hyperthymic and cyclothymic temperaments.

Finally, we did not use specific questionnaires to assess eating disorders, such as compulsive eating disorder.

## CONCLUSIONS

In conclusion, in the age group of 50 years or more, cases of morbid obesity have a significantly greater chance (2.56 times) of occurring in individuals with a hyperthymic temperament, compared to individuals who do not have this temperament. Among the three types of affective temperaments evaluated, only hyperthymia could be a risk factor for morbid obesity.
